# Metagenomic analysis of microbial consortia native to the Amazon, Highlands, and Galapagos regions of Ecuador with potential for wastewater remediation

**DOI:** 10.1111/1758-2229.13272

**Published:** 2024-05-01

**Authors:** Juan José Guadalupe, Miguel Pazmiño‐Vela, Gabriela Pozo, Wendy Vernaza, Valeria Ochoa‐Herrera, Maria de Lourdes Torres, Andres F. Torres

**Affiliations:** ^1^ Laboratorio de Biotecnología Vegetal, Colegio de Ciencias Biológicas y Ambientales Universidad San Francisco de Quito (USFQ), Calle Diego de Robles y Avenida Pampite Quito Ecuador; ^2^ Colegio de Ciencias e Ingeniería Universidad San Francisco de Quito USFQ, Diego de Robles y Vía Interoceánica Quito Ecuador; ^3^ Department of Environmental Sciences and Engineering, Gillings School of Global Public Health University of North Carolina at Chapel Hill Chapel Hill North Carolina USA

## Abstract

Native microbial consortia have been proposed for biological wastewater treatment, but their diversity and function remain poorly understood. This study investigated three native microalgae‐bacteria consortia collected from the Amazon, Highlands, and Galapagos regions of Ecuador to assess their metagenomes and wastewater remediation potential. The consortia were evaluated for 12 days under light (LC) and continuous dark conditions (CDC) to measure their capacity for nutrient and organic matter removal from synthetic wastewater (SWW). Overall, all three consortia demonstrated higher nutrient removal efficiencies under LC than CDC, with the Amazon and Galapagos consortia outperforming the Highlands consortium in nutrient removal capabilities. Despite differences in α‐ and β‐diversity, microbial species diversity within and between consortia did not directly correlate with their nutrient removal capabilities. However, all three consortia were enriched with core taxonomic groups associated with wastewater remediation activities. Our analyses further revealed higher abundances for nutrient removing microorganisms in the Amazon and Galapagos consortia compared with the Highland consortium. Finally, this study also uncovered the contribution of novel microbial groups that enhance wastewater bioremediation processes. These groups have not previously been reported as part of the core microbial groups commonly found in wastewater communities, thereby highlighting the potential of investigating microbial consortia isolated from ecosystems of megadiverse countries like Ecuador.

## Graphical abstract

This study explores the metagenomes and wastewater treatment potential of native microalgae–bacteria consortia from Ecuador's Amazon, Highlands, and Galapagos regions under both light and continuous dark conditions. All consortia displayed higher nutrient removal efficacies under light, with the Amazon and Galapagos consortia outperforming the Highlands consortium. While the three consortia contained core taxonomic groups characteristic of wastewater communities, our research unveiled novel microbial groups, emphasizing the potential of megadiverse ecosystems as reservoirs of microbial consortia essential for wastewater treatment.

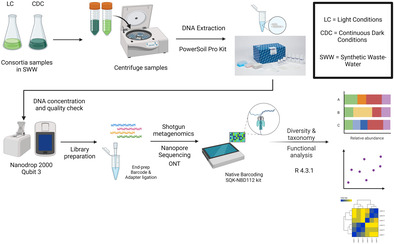

## INTRODUCTION

Anthropogenic activities produce significant amounts of wastewater carrying a broad range of inorganic and organic pollutants. Globally, wastewater is routinely discharged into freshwater resources without adequate treatment (2017 UN World Water Development Report, Wastewater: The Untapped Resource | UNEP – UN Environment Programme, n.d.). This continuous discharge results in oxygen depletion and the collateral degradation of freshwater ecosystems through the loss of biodiversity (Beman et al., 2005; Diaz & Rosenberg, 2008; Heisler et al., 2008; Smith, 2003; Smith et al., 1999). The progressive enrichment of freshwater resources with inorganic and organic pollutants may also lead to the accumulation of cyanobacteria (or other microorganisms) that produce hazardous toxins that compromise the safety of drinking water and aquatic food supplies (Codd, 2000; Paerl et al., 2020). The discharge of untreated wastewater clearly poses critical challenges to the environment and public health. Therefore, there is an urgent need for cost‐effective, efficient, and environmentally compatible technologies for wastewater treatment (WWT).

In the developing world, the primary challenge in wastewater management is the development of robust, practical and easy to operate treatment systems that can degrade organic matter (e.g., carbohydrates, fats, proteins) and remove inorganic nutrients—specially nitrogen (N) and phosphorous (P) (Gallego‐Schmid & Tarpani, 2019). Innovations in this direction are crucial to achieve the United Nations (UN) Sustainable Development Goal no. 6 “Clean Water and Sanitation” by 2030 (2017 UN World Water Development Report, Wastewater: The Untapped Resource | UNEP – UN Environment Programme, n.d.). Existing tertiary treatment strategies for the removal of N, P, and organic matter (e.g., anaerobic digestion followed by biological denitrification, chemical precipitation) tend to be technically complex, entail high implementation costs, and are energy demanding (Lee & Lei, 2019; Leflay et al., 2020; Quijano et al., 2017; Zhang et al., 2021). In this respect, wastewater remediation using microalgae‐bacteria consortia has emerged as a cost‐effective strategy (Abdelfattah et al., 2023). Photosynthetic microalgae are autotrophic organisms that require large amounts of N and P for their growth, which makes them ideal for the uptake of nutrients from wastewater (Renuka et al., 2013). The nutrient removal capacity of microalgae is further enhanced by their cooperative metabolic interactions with co‐cultivated bacteria. For example, photosynthetic microalgae produce dissolved oxygen to drive bacterial growth, and in turn, bacteria supply essential secondary metabolites and supplementary inorganic carbon (through the breakdown of organic matter) to stimulate microalgae growth through respiration (Fallahi et al., 2021; Zhang et al., 2021). Ultimately, the cultivation of microalgae–bacteria consortia using wastewater is an efficient, biologically driven nutrient removal system with low implementation costs, easy scalability, and low energy demand (Lee & Lei, 2019; Leflay et al., 2020).

Multiple studies have established that microalgae–bacteria consortia are effective at removing N, P, and organic matter from wastewater (Jia & Yuan, 2016; Sátiro et al., 2022; Zhang et al., 2021). The species composition of the consortia is an important factor, as this leads to different remediation efficacies (Liu et al., 2017). Bacterial and microalgal species differ greatly in growth rate, nutrient removal capacity, and preferred habitat (Liu et al., 2017). For instance, certain species, such as the filamentous microalgae *Cladophora* spp., *Klebsormidium* spp., and *Pseudanabaena* spp., as well as bacteria of the genus Accumulibacter and Pseudomonas, are naturally more effective removers of N and P (Liu et al., 2017; Liu & Vyverman, 2015). In this context, native (or indigenous) consortia, as opposed to synthetic monocultures or composite strains, may demonstrate greater ability to remediate wastewater in their native environments (Benítez et al., 2019; Choudhary et al., 2016; Gonçalves et al., 2017). Since native consortia already grow in the environment to be remediated, these show greater resilience, adaptation, and aptitude to the natural range of temperature, irradiance, pH, and other (a)biotic factors that characterize the target environment (Liu et al., 2017). A better understanding of the remediation capabilities of native microalgae–bacteria consortia under both light and dark conditions promises to uncover new approaches for designing effective in situ and ex situ wastewater remediation strategies. For instance, cultivating microalgae–bacteria consortia in wastewater with limited light (i.e., by exploiting the heterotrophic or mixotrophic growth habits of algae and cyanobacteria) could enable the design of photobioreactors (PBRs) that require significantly less land and energy for efficient operation (Fan et al., 2020; Zhang et al., 2013).Additionally, understanding the metabolic capabilities of diverse native consortia offers insights into engineering microbial communities with improved robustness and nutrient degradation efficiency in industrial settings (Johnson & Admassu, 2013).

Ecuador ranks among the 17 most megadiverse countries globally, boasting the highest biodiversity per square kilometre (Mestanza‐Ramón et al., 2020; Negru et al., 2020). Despite its extensive biodiversity, much of Ecuador's microbial diversity remains unexplored, presenting opportunities for the discovery of microorganisms with remediation potential. For instance, Ecuador's biodiversity has yielded valuable microbial consortia such as *Lentinus* spp., *Bacillus* spp., and *Geomyces* spp., sourced from the Ecuadorian Amazon. These microorganisms have demonstrated remarkable efficacy in remediating effluents contaminated with heavy metals, hydrocarbons, and petroleum, respectively (Maddela et al., 2015; Osório da Rosa et al., 2022).

The objective of this study was to characterize the microbial composition of microalgae–bacteria consortia native to the Amazon, Highlands, and Galapagos regions of Ecuador, and to investigate correlations between microbial composition and wastewater remediation efficacy. We analysed the capacity of these consortia to remove nitrogen, phosphorus, and organic matter from SWW through removal bioassays conducted under both light (LC) and continuous dark conditions (CDC). This approach aimed to determine if light influences the bulk nutrient removal efficacy of the evaluated consortia in wastewater environments. Additionally, we examined the metagenomic composition of the three consortia during the nutrient removal bioassays to identify correlations between taxonomic and functional abundances and the removal efficacies of the evaluated consortia. This study represents a crucial initial step in characterizing the microbial diversity present in megadiverse regions like Ecuador, particularly in terms of their potential for wastewater remediation.

## EXPERIMENTAL PROCEDURES

### Consortia sampling and propagation

**FIGURE 1:**
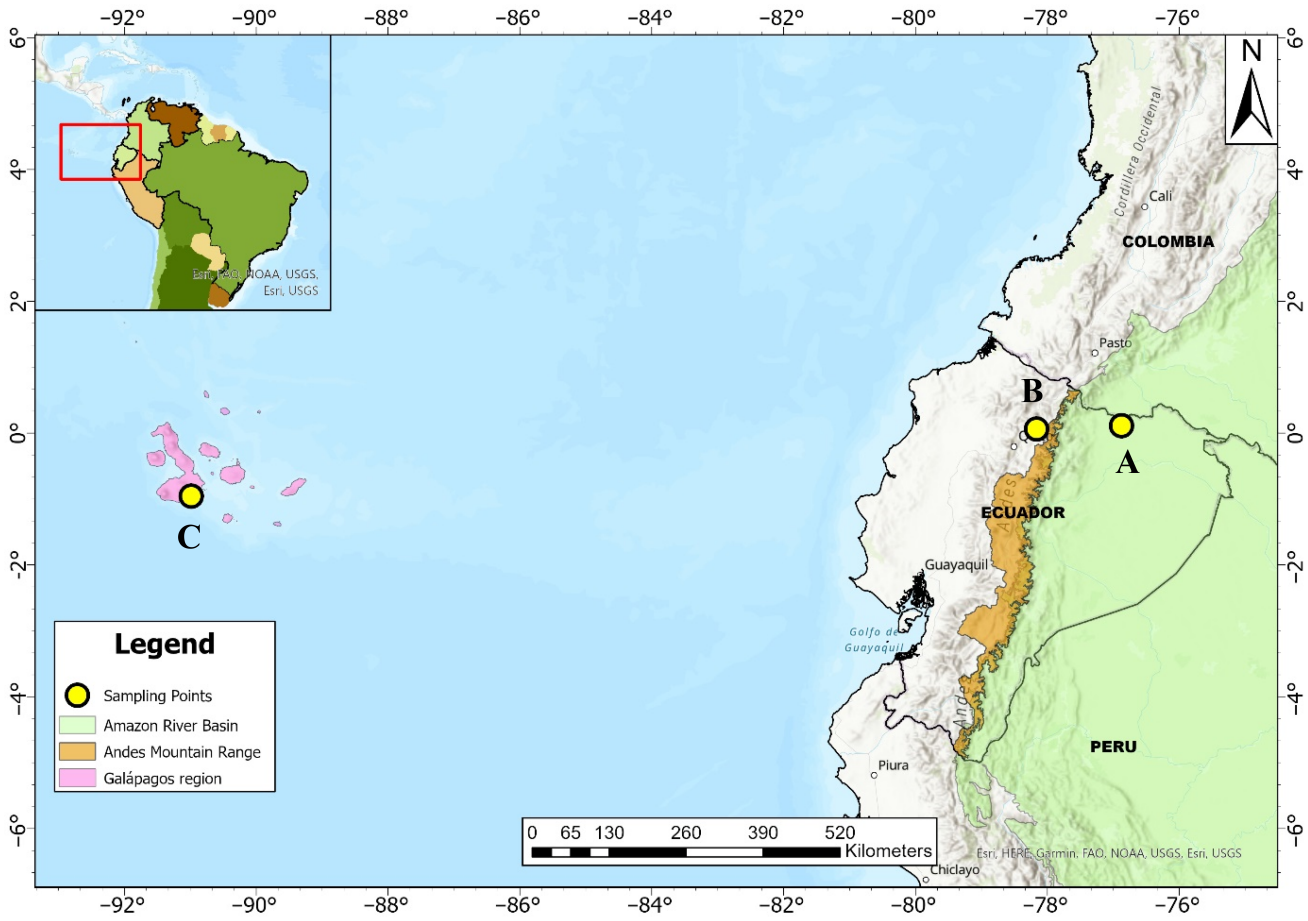
Map of Ecuador indicating the sampling sites for each of three microalgae–bacteria consortia native to the (A) Amazon, (B) Highlands, and (C) Galapagos regions of Ecuador. The map was generated using ArcGIS Pro 3.2.

In this study, we assessed the ability of three microalgae–bacteria consortia, collected from the Amazon, Highlands, and Galapagos regions of Ecuador, to remove nutrients and organic matter from wastewater. Figure 1 illustrates the collection sites of the three evaluated consortia.

To build up sufficient inoculum for the nutrient removal bioassays, the three microalgae–bacteria consortia were independently propagated in SWW using a two‐step scale‐up process. In the first scale‐up phase, 2.5 mL of inoculum was aseptically added to 47.5 mL SWW in a 50 mL glass flask. SSW composition in g/L was: 0.2905 CaCl_2_ · 2H_2_O, 0.089 MgSO_4_ · 7H_2_O, 0.5121 (NH_4_)_2_CO_3_, 0.0001 NaNO_3_, 0.0454 KH_2_PO_4_, 0.0003 FeSO_4_ · 7H_2_O, 0.0001 ZnCl_2_, 0.0002 CuSO_4_ · 5H_2_O, 0.8256 C_6_H_12_O_6_, and 1 mL/L trace metals solution. SWW pH was adjusted to 7.0 (±0.06) using 1 M HCl. Before use, SWW was filtered using sterile Whatman® PURADISC 0.45 μm microfiber cellulose filters (Merck, Germany). Glass flasks were sealed with sterile caps made from cotton and gauze and were incubated at room temperature (23°C ± 0.5°C) for 15 days using a 12‐h photoperiod under constant agitation (114 rpm). Artificial light was provided using 20 W OSRAM tubular fluorescent lamps and LED lights (91.8 μmol/m^2^/s). After the 15‐day incubation period, a second scale‐up phase was initiated by adding 50 mL of the cultivated consortia to 450 mL SWW in new 500 mL glass flasks. Glass flasks were aseptically sealed and incubated for an additional 15 days using the same temperature, agitation, photoperiod, and light conditions used during the first scale‐up phase. After the second scale‐up process, cellular densities for the consortia ranged from 0.8 to 1.0 g/L; sufficient to conduct nutrient removal bioassays.

### Nutrient removal bioassays

#### PBR setup and operational conditions

For the three consortia, nutrient removal bioassays were conducted in duplicate under 12 h‐light (LC) and continuous dark conditions (CDC) and they were composed of 70 mL of propagated microalgae–bacteria consortia (produced during the second scale‐up propagation process) and 930 mL membrane‐filtered SWW. Abiotic (AB) and heat‐killed inoculum (HK) controls were also included and were performed in duplicate. HK control bioassays were prepared by combining 70 mL (autoclave‐inactivated) inoculum with 930 mL membrane‐filtered SWW. AB control bioassays were prepared by combining 70 mL Milli‐Q® water (Merck Millipore, Massachusetts) with 930 mL membrane‐filtered SWW. All bioassays were carried out in SWW (refer to Section 2.1) in PBRs consisting of a total initial volume of 1000 mL in Erlenmeyer flasks sealed with autoclaved caps made of gauze and cotton and all PBRs were incubated for 12 days at room temperature (23°C ± 0.5°C) under continuous agitation (114 rpm). Throughout the experiment, PBRs were supplemented with artificial light using tubular 20 W OSRAM fluorescent lamps and LED lights (91.8 μmol/m^2^/s) using a 12 h photoperiod. CDC bioassays were assembled and incubated identically to LC PBRs but were completely covered with aluminium foil to prevent the PBRs from receiving any light.

#### Analyses of nutrient and organic matter removal

For all bioassays (and controls), a 10 mL sample was aseptically collected on Day 0 (D0), Day 6 (D6), and Day 12 (D12) to evaluate nutrient concentrations (NH_4_^+^, PO_4_^3−^), chemical oxygen demand (soluble COD; used as a proxy for organic matter concentration), and pH. Standard protocols were used for all analyses (American Public Health Association et al., 2023). Ammonium (NH_4_^+^; SM 4500‐NH3‐D) and pH were determined potentiometrically using a Thermo Scientific Orion 5‐Star portable multi‐parameter (Thermo Specific Ion Selective Electrode, ISE Orion). Phosphate (PO_4_^3−^; SM 4500‐P‐B) and soluble COD (SM 5520‐B) were determined calorimetrically using a visible spectrophotometer (Thermo Scientific Inc. GENESYS 30, USA). For phosphate, the principle of colorimetric analysis involves the reaction of orthophosphate with ammonium molybdate and potassium antimonyl tartrate in an acidic medium to form a heteropoly acid‐phosphomolybdic acid, which is then reduced to coloured molybdenum blue by ascorbic acid (American Public Health Association et al., 2023). For the COD colorimetric analysis, the principle entails the use of dichromate ion as an oxidant to promote the oxidation of organic matter present in the bioassays (American Public Health Association et al., 2023).

The efficacy of a consortium in removing nutrients and organic matter (COD) from SWW was assessed by calculating the percentage of bulk NH_4_^+^, PO_4_^3−^, or COD removed by D12 relative to the initial concentration of the respective nutrient at D0. For each consortium and treatment, bulk nutrient removal (%) levels were respectively calculated per duplicate batch bioassay using the following formula:

$$\text{Bulk nutrient removal}\;\left(\%\right)=\frac{C_{f}-C_{0}}{C_{0}}\times 100,$$

where *C*_0_ is the initial concentration on D0 and *C*_0_ is the final concentration on D12.

For each treatment (LC and CDC), one‐way analysis of variance (ANOVA) with a least significant difference (LSD) test was conducted to determine significant differences in the removal efficacies of the three consortia at D6 and D12. Per each consortium and treatment combination, differences in nutrient concentration levels between sampling dates were assessed using a paired *t*‐test (*p* < 0.05), and differences in nutrient concentrations relative to the AB and HK controls on D6 and D12 were assessed using a two‐sample *t*‐test (*p* < 0.05). Per consortium, significant differences in bulk nutrient removal (%) under LC and CDC were also confirmed using a two‐sample *t*‐test. All statistical analyses were performed in R.

### Shotgun metagenomic analyses

#### DNA sampling and genomic sequencing

For all consortia, shotgun metagenomic analyses were performed for LC and CDC samples collected on D0, D6, and D12 of the nutrient removal bioassays. A 10 mL sample was taken from the PBR at each timepoint. Collected samples were centrifuged at 9000 RPM for 8.5 min to concentrate the microbial biomass. After discarding the supernatant (which was used to evaluate nutrient concentrations, COD, and pH), the concentrated microbial biomass was transferred to a mortar and triturated and homogenized with liquid nitrogen. DNA from collected samples was isolated using the Qiagen DNeasy PowerSoil Pro kit (Qiagen) according to the manufacturer's instructions. DNA quality and concentration were assessed using a NanoDrop 2000 (ThermoFisher) and Qubit 3 (Invitrogen). DNA was purified using AMPure XP beads at a concentration of ×1.8 (Beckman‐Coulter).

DNA samples from each consortium and each treatment (LC/CDC) were independently barcoded using the Oxford Nanopore Technologies (ONT) Native Barcoding SQK‐NBD112 kit according to the manufacturer's instructions. Barcoded samples were pooled per timepoint to create three unique libraries. Sequencing libraries were prepared using the ONT Ligation Sequencing Kit LSK‐109 following the manufacturer protocols. The three libraries were run on independent FLOMIN106 Flow Cells (R9.4) in a Minion Mk1b device for 24 h. Base calling was carried out in real time using the MinKNOW software (v. 20.10.3).

#### Bioinformatic analyses

After sequencing, raw reads were processed with Porechop v0.2.3 (Wick et al., 2017) to remove adaptors and barcodes. Human DNA contaminants were eliminated using an in‐house script based on BWA‐MEM, utilizing the GRCh38 human genome as a reference. Nanoplot 1.38.1 (De Coster & Rademakers, 2023) was employed to extract and visualize sequence quality parameters.

For taxonomic assignments, the co‐assembly function of SqueezeMeta v1.3.0 (Tamames & Puente‐Sánchez, 2019) was utilized, enabling querying, mapping, and estimation of sequence abundances against the GenBank database. To investigate microbial community composition, the relative abundance of taxonomic units at the phylum level was calculated for each consortium on D0, D6, and D12 under LC and CDC. These results were then visualized using the SQMtools_1.6.2 R package (Puente‐Sánchez et al., 2020). To ensure comparability, all sequences were rarefied to a standardized depth of 12,000 sequences per sample, based on the sample with the lowest sequencing depth. Rarefaction was performed using an in‐house custom script in R 4.2.1. Linear discriminant analysis effect size (LEfSe) was used to identify and compare the most prevalent microbial groups present in each consortium (Segata et al., 2011). Taxonomic groups with LDA values higher than 2 at a *p*‐value <0.05 were considered statistically significant. LEfSe was conducted online in the Galaxy workflow framework (Afgan et al., 2022; Segata et al., 2011), and visualization of LEfSe differences between the three consortia were accomplished using the ggplot2 R package (Wickham, 2016).

Shannon, inverse Simpson, and Evenness α‐diversity indices were calculated using the Vegan v2.6.2 R Package (Oksanen et al., 2015) using rarefied taxonomic units. For β‐diversity analysis, Bray–Curtis distances based on taxonomic abundance profiles were used. Permutational multivariate analysis of variance (PERMANOVA) was applied to measure the statistical significances of β‐diversity. The β‐diversity analyses were performed using the Ohchibi R Package (Salas Gonzalez, 2019).

We used SqueezeMeta v1.3.0 to predict the functional gene content of the three consortia under LC and CDC. Predicted gene functions were annotated using the KEGG pathway database and investigated through two distinct approaches (targeted vs. untargeted). In the targeted approach, we focused on genes specifically involved in nitrogen, phosphate, carbohydrate, and photosynthesis metabolism. These metabolic activities were deemed relevant to the degradation, conversion, or assimilation of nutrients (NH_4_^+^ and PO_4_^3−^) and organic matter (COD) in SWW (Ahmad et al., 2021; Ojha et al., 2021). In the untargeted approach, we examined genes that demonstrated a high correlation (*r* ^2^ > 0.7) with NH_4_^+^, PO_4_^3−^, and COD removal patterns, based on the square of the Pearson product moment correlation coefficient. In both approaches, we calculated the relative abundance of predicted genes for each consortium on D0, D6, and D12 under LC and CDC. Subsequently, we visualized the results as heatmaps using the SQMtools_1.6.2 R package.

## RESULTS

### Nutrient removal bioassays

#### General removal patterns

Three microalgae–bacteria consortia collected from distinct regions of Ecuador—Amazon, Highlands, and Galapagos—were cultivated for 12 days in SWW. Their bulk nutrient removal capacity for NH_4_^+^, PO_4_^3−^, and organic matter (measured as soluble COD) were assessed under light (LC) and continuous dark conditions (CDC) and are presented in Table 1. The final nutrient degradation levels for the respective abiotic (AB) and heat‐killed inoculum (HK) controls are also shown in Table 1.

**TABLE 1:** Nutrient bulk removal efficacies (measured as percentage of nutrient/organic matter removed from initial concentrations on Day 0 compared with final concentrations on Day 12) for ammonium (NH_4_^+^), phosphate (PO_4_^3−^), and soluble COD for three consortia from the Ecuadorian Amazon, Highlands, and Galapagos regions.

Consortia	Bioassay	Removal efficacy (%)		
		NH_4_^+^	PO_4_^3−^	COD
Amazon	AB	9.1	3.7	6.1
	HK	3.3	9.0	9.5
	CDC	41.0	64.5	85.9
	LC	55.3	83.2	97.6
Galapagos	AB	4.8	0.0	3.0
	HK	6.8	5.0	4.5
	CDC	29.9	53.1	70.5
	LC	41.2	89.9	82.2
Highlands	AB	3.0	9.0	1.9
	HK	5.0	9.7	9.7
	CDC	12.0	41.1	50.9
	LC	30.4	77.2	60.4

For all consortia, NH_4_^+^, PO_4_^3−^, and COD concentrations on D6 and D12 under LC and CDC were significantly lower (paired *t*‐test, *p* < 0.001) than nutrient concentrations on D0, and consistently lower (two‐sample *t*‐test, *p* < 0.001) than nutrient concentrations observed for the AB and HK controls on D6 and D12. For the AB and HK controls, nutrient degradation on D12 was marginal (<10%).

Under LC, the bulk removal (%) efficacies of the three consortia were significantly higher (two‐sample *t*‐test, *p* > 0.05) than those observed under CDC. On average, bulk removal (%) capacity for NH_4_^+^, PO_4_^3−^, and COD improved by 75%, 62%, and 16%, respectively, under LC compared with CDC. Throughout the bioassays, the pH of the reaction medium showed an increasing trend, rising from 7.0–7.5 on D0 to ~8.2–8.7 by D6 and remained stable until D12 (Figure S1).

#### Consortia‐level differences in nutrient removal efficiencies

**FIGURE 2:**
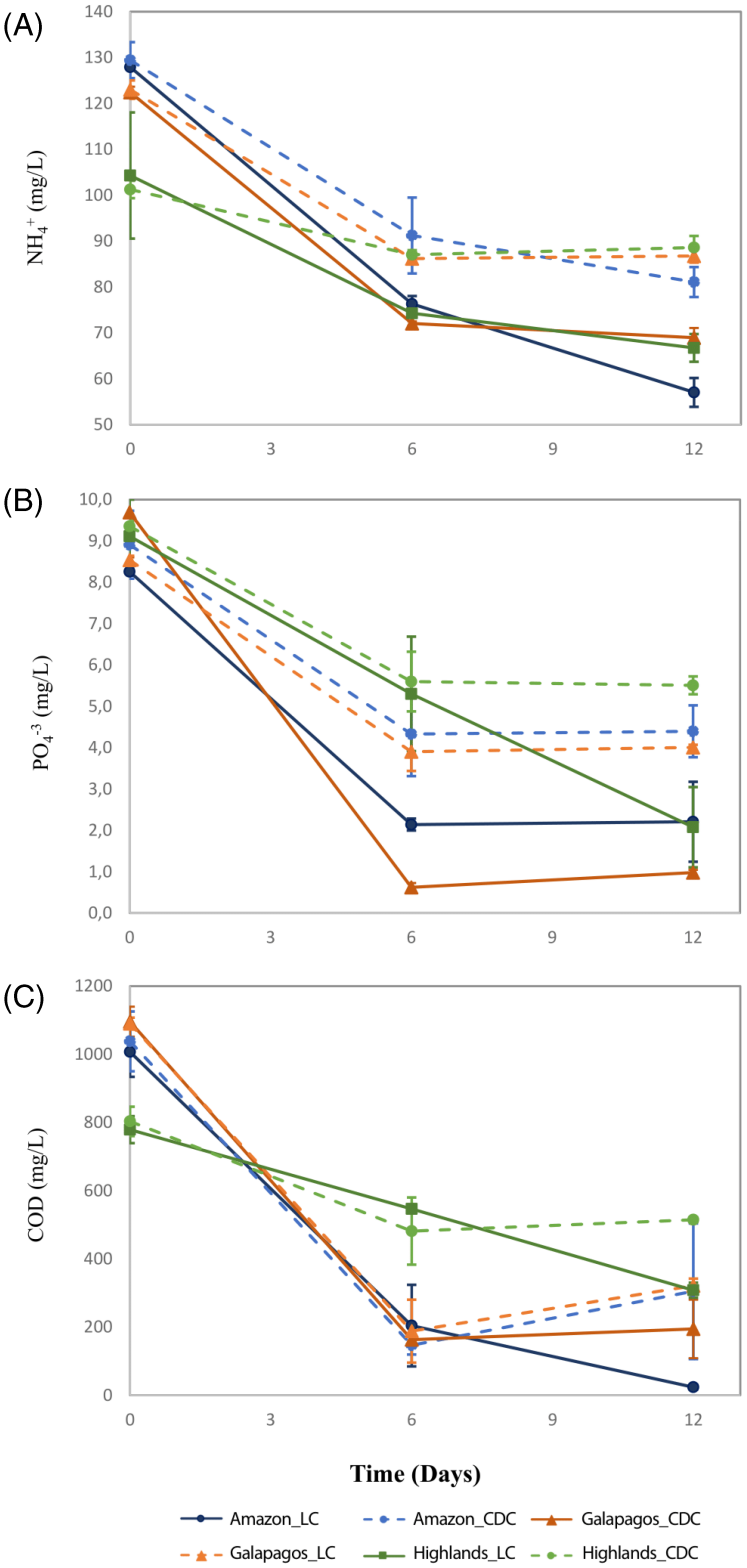
Nutrient removal patterns for (A) ammonium (NH_4_^+^), (B) phosphate (PO_4_^3−^), and (C) soluble COD for three microalgae‐bacteria consortia from the Ecuadorian Amazon, Highlands, and Galapagos regions incubated in SWW under light (LC) and continuous dark (CDC) conditions. All batch bioassays were carried out in duplicate. Results show means and error bars indicate ± standard deviation of duplicate measures.

Figure 2 illustrates the nutrient removal trends for the three consortia over time under LC and CDC. Across all three consortia and treatment conditions, the removal of NH_4_^+^, PO_4_^3−^, and COD exhibited a similar pattern. This general pattern was characterized by a significant decrease in SWW nutrient concentration from D0 to D6, followed by a moderate‐to‐low decline in nutrient concentration for the remainder of the incubation period. To illustrate, for the Galapagos consortium under LC, NH_4_^+^ concentration decreased by 41% from an initial concentration of 122 mg/L on D0 to 72 mg/L by D6. By the end of the experiment (D12), the concentration of NH_4_^+^ decreased by an additional 4% to 69 mg/L compared with the concentration on D6. Deviation from this general trend was observed for the Highlands consortium regarding the removal of PO_4_^3−^ and COD under LC, and for the Amazon consortium regarding the removal of COD under LC. In these cases, the degree of nutrient degradation from D6 to D12 was (significantly) higher than the degree of nutrient degradation observed from D0 to D6. In particular, the Highlands consortium showed a ~1.45‐fold increase in removal efficacy from D6 to D12 (61% for PO_4_^3−^; 43% for COD) relative to the removal efficacy observed from D0 to D6 (42% for PO_4_^3−^; 30% for COD).

When comparing the bulk removal efficacies of the three consortia under LC and CDC, the Highlands consortium consistently and significantly (ANOVA, *p* < 0.05) underperformed the Amazon and Galapagos consortia in removing NH_4_^+^, PO_4_^3−^, and COD (Table 1). Bulk removal efficiencies for the Highlands consortium were on average ~1.3‐fold lower for PO_4_^3−^ and COD, and ~1.6‐fold lower for NH_4_^+^. Between the Amazon and Galapagos consortia, the former generally exhibited higher bulk removal efficiencies (ANOVA, *p* < 0.05) under LC and CDC, except for PO_4_^3−^ under LC, where the Galapagos consortium showed statistically superior efficiency, and COD under CDC, where the two consortia did not show statistically significant differences. Notably, while the Amazon and Galapagos consortia displayed similar removal patterns (i.e., sharp removal efficacy from D0 to D6, followed by qualitatively lower removal rates from D6 to D12), the Amazon consortium generally showed higher removal efficiencies from D6 to D12 under LC (21% higher for NH_4_^+^; 88% higher for COD) and CDC (11% higher for NH_4_^+^).

### Metagenomic analyses

#### Microbial community structure

To investigate the composition of microbial communities, we conducted a metagenomic analysis of the three consortia using nanopore sequencing. Sequencing statistics are provided in Table S1. The percentage of unclassified reads across all consortia, treatments, and sampling points ranged from 34% to 81%. Among the three consortia, the Galapagos consortium had the highest number of unclassified reads (mean = 68%), followed by the Amazon (mean = 61%) and Highlands consortia (mean = 40%).

**FIGURE 3:**
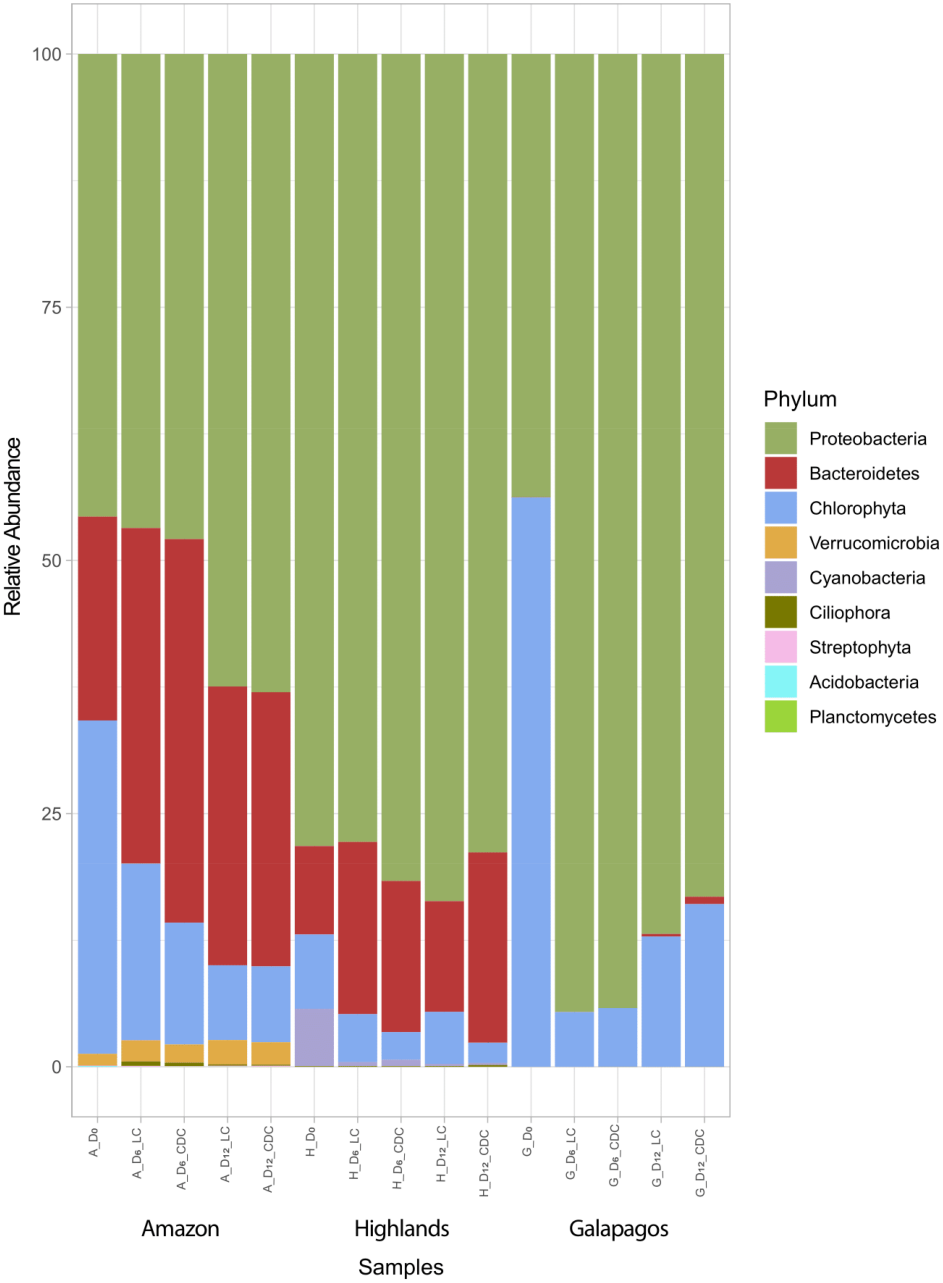
Percent relative abundance of the most represented taxa (phylum level) for three microalgae–bacteria consortia from the Ecuadorian Amazon, Highlands, and Galapagos regions incubated in SWW under light (LC) and continuous dark (CDC) conditions.

Figure 3 illustrates the microbial community composition at the phylum level for the three consortia under LC and CDC over time. The predominant phyla for the Highlands and Amazon consortia, across all sampling points and treatments, were Proteobacteria, Chlorophyta, and Bacteroidetes. The Galapagos consortium also showed Proteobacteria and Chlorophyta as the predominant phyla, but Bacteroidetes had a negligible presence. Notably, while the Highlands consortium had a significant presence of Cyanobacteria (7.4%) at D0, this phylum was minimally represented (<2.1%) on D6 and D12 in all three consortia under LC and CDC.

In general, microbial community compositions were stable over time and between treatments. For example, Proteobacteria exhibited stable relative abundances from D0 to D12 for the Highlands (79%–84%) and Amazon (44%–54%) consortia under LC and CDC. The only significant deviation from this trend was observed in the Galapagos consortium, which showed a sharp increase in the abundance of Proteobacteria from D0 (43%) to D6 (95%) under LC and CDC, stabilizing by D12 at 86% under LC and 80% under CDC. It is also worth noting that for all three consortia, the abundance of Chlorophyta decreased (to varying degrees) from D0 to D6 under LC and CDC. However, while Chlorophyta continued to decrease in abundance from D6 to D12 under LC and CDC in the Amazon and Highlands consortia, the Galapagos consortium showed an increase in the abundance of Chlorophyta from D6 to D12 under LC and CDC.

**FIGURE 4:**
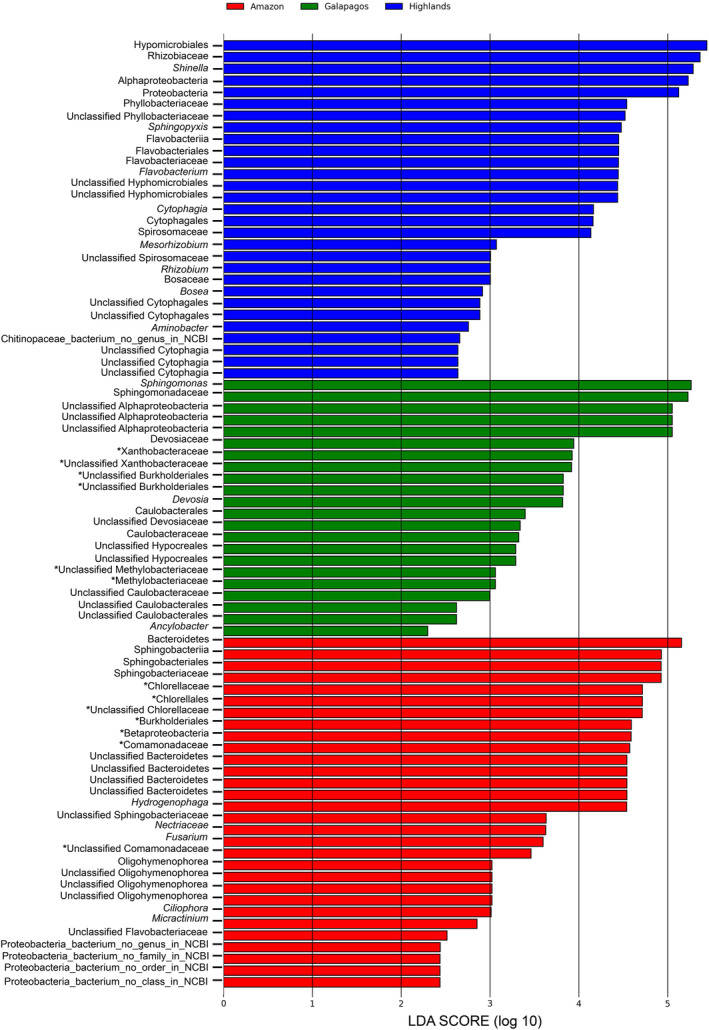
Histogram showing linear discriminant analysis (LDA) scores for the most abundant taxonomic groups (genus level) in three microalgae–bacteria consortia from the Ecuadorian Amazon, Highlands, and Galapagos regions incubated in SWW under light (LC) and continuous dark (CDC) conditions. Significance was tested by Kruskal–Wallis test (*α* < 0.05) and log LDA score (>2.0). Asterisks (*) highlight microbial taxa commonly found in wastewater communities (Dueholm et al., 2022; Wu et al., 2019).

To further investigate the microbial communities of the three consortia, we used linear discriminant analysis effect size (LEfSe) to identify differentially abundant taxa. This analysis discovered 62 taxonomic groups which were significantly abundant (LDA > 2.0, *p* < 0.05) and discriminative between the three consortia (Figure 4). For all three consortia, several of these taxonomic groups have been associated with remediation functions in wastewater treatment plants (Dueholm et al., 2022; Meng et al., 2016; Moloantoa et al., 2023; Tikhonova et al., 2021; Tsagkari & Sloan, 2019; Wu et al., 2019). From these taxonomic groups, the Amazon consortium displayed a higher abundance (LDA > 3) of the Bacteroidetes and Comamonadaceae families, as well as the Hydrogenophaga and β‐proteobacteria genera. In the Galapagos consortium, the Devosiaceae, Xanthobacter, Caulobacter, Burkholderiales and Methylobacter taxonomic groups were significantly more enriched (LDA > 3) than in the other two consortia. Meanwhile, the Highlands consortium had an increased abundance of Hyphomicrobiales and Flavobacterium. Notably, the genus Sphingomonas appeared to be abundant in all three consortia, indicating its wide distribution and potential ecological significance.

#### α‐ and β‐diversity analyses

Shotgun metagenomic analysis was used to determine α‐ and β‐diversity indices for the three evaluated consortia. Tables 2 and S2 show α‐diversity indexes (Shannon, Inverse Simpson, Evenness) for the three consortia across time under LC and CDC, respectively. On average, Shannon indices (D_Shannon_) did not vary widely across the three consortia or across time. In contrast, Inverse Simpson indices showed qualitatively greater differences across the three consortia and across time. For instance, the Amazon consortium showed the highest degree of within‐group diversity (D_Inv‐Simpson_ = 6.39–9.79) compared with the Galapagos consortium (D_Inv‐Simpson_ = 2.46–4.29) and the Highlands consortium (D_Inv‐Simpson_ = 5.16–5.42) under LC throughout the entire incubation period. Accordingly, while D_Inv‐Simpson_ for the Amazon consortium increased 1.5‐fold from D0 to D12 (Table 2), D_Inv‐Simpson_ decreased by 1.2‐fold from D0 to D12 for the Galapagos consortium and remained virtually unchanged for the Highlands consortium throughout the nutrient bioassay. Inverse Simpson indices under CDC and Evenness indices under LC and CDC followed a similar pattern as described above for the three consortia under LC. Pearson correlation analyses showed no significant correlations between nutrient degradation patterns and α‐diversity indices over time.

**TABLE 2:** α‐Diversity indices at different timepoints for three Ecuadorian microbial consortia from the Amazon, Highlands, and Galapagos regions incubated in SWW for a 12‐day period under light (LC) conditions.

		α‐Diversity index		
Consortia	Timepoint	Shannon	Inverse Simpson	Evenness
Amazon	D0	2.34	6.39	0.57
	D6	2.58	9.79	0.63
	D12	2.62	9.57	0.62
Galapagos	D0	1.85	4.29	0.47
	D6	1.57	2.46	0.4
	D12	1.76	3.58	0.45
Highlands	D0	2.31	5.42	0.54
	D6	2.29	5.54	0.55
	D12	2.28	5.16	0.55

**FIGURE 5:**
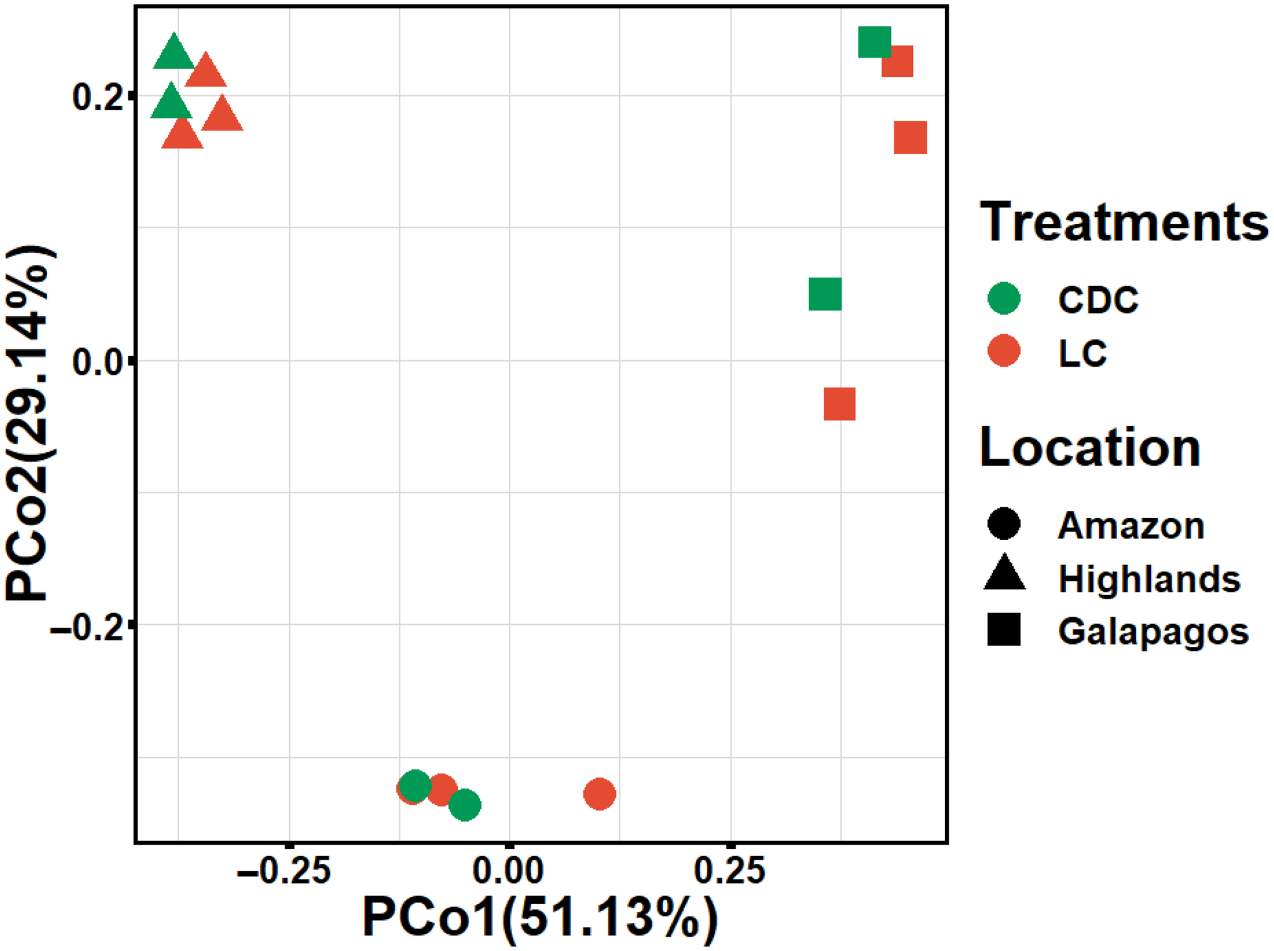
Principal coordinate analyses of Bray–Curtis beta‐diversity showing the effects of geographic origin (Location) and light conditions (Treatments) on the microbial community structure of three microalgae–bacteria consortia from the Ecuadorian Amazon, Highlands, and Galapagos regions incubated in SWW under light (LC) and continuous dark (CDC) conditions.

A beta diversity dissimilarity matrix was generated using Bray–Curtis distances between all samples and examined using Principal Coordinate Analysis (PCoA). Axes 1 and 2, representing 47.1% and 32.4% of the variance, respectively, are displayed in Figure 5. The PCoA plot shows good clustering for all samples (i.e., across treatments and sampling points) belonging to the same consortium, and Bray–Curtis distances revealed significant (*p* < 0.001) community‐level separation between the three consortia. It is relevant to highlight that greater variance and lower cluster definition was observed for samples belonging to the Amazon consortium (Figure 5). Treatment (LC, CDC) and treatment‐by‐consortia origin interactions did not have a significant effect on microbial‐community level differences between the three consortia.

#### Predicted functions

**FIGURE 6:**
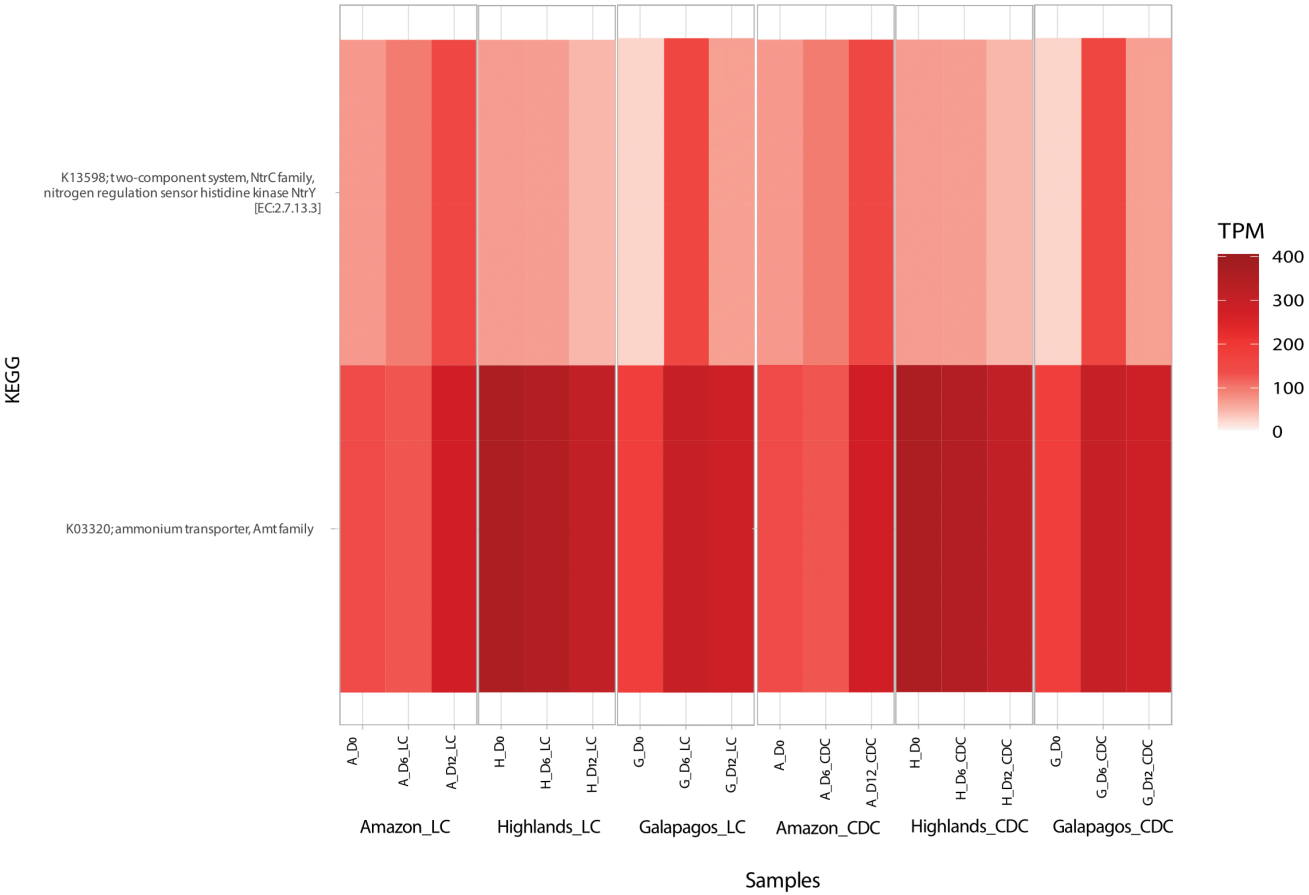
Heatmap showing the relative abundance for the two most dominant KEGG pathway identifiers (KP IDs) associated with nitrogen metabolism (i.e., ammonium metabolism and nitrogen fixation) for three Ecuadorian microalgae–bacteria consortia from the Ecuadorian Amazon, Highlands, and Galapagos regions incubated in SWW under light (LC) and continuous dark (CDC) conditions. Transcripts per million (TPM) refers to the number of genes from each KEGG pathway per million genes in the metagenome.

To gain insights into the complex metabolism of the three consortia during SWW remediation, we evaluated their functional gene content over time under LC and CDC. This analysis revealed 112 predicted genes (KP IDs) associated with nitrogen, phosphorous, carbohydrate, and photosynthesis metabolism, of which 30% were common to all consortia (Table 3). For instance, all predicted genes identified for ammonium degradation (K11741: Quaternary ammonium compound; K03320: Ammonium transporter Amt family) (Figure 6) and 60% of all predicted genes related to photosynthesis were shared by the three consortia across all treatments and timepoints (Figure S2). For nitrogen fixation, phosphate metabolism, and carbohydrate metabolism, the shared percentage of predicted genes was considerably lower (respectively, 17%, 14%, and 26%), but the three consortia shared the most abundant KP ID for each of these metabolic pathways across all treatments and timepoints (i.e., K13598 for nitrogen fixation, K01652 for phosphate metabolism, and K00249 for carbohydrate metabolism). It is worth noting that the three consortia showed unique relative abundance patterns for the shared predicted genes across timepoints and treatments. This made it particularly challenging to infer correlations between predicted gene enrichment patterns specifically associated with nitrogen, phosphorous, carbohydrate, and photosynthesis metabolism and nutrient removal trends.

**TABLE 3:** Percentage of KEGG pathway IDs shared by three microbial consortia from the Amazon, Highlands, and Galapagos regions of Ecuador incubated in SWW for a 12‐day period.

KEGG pathway	#KP IDs identified	% KP IDs similar for all consortia
Nitrogen metabolism (Ammonium)	2	100%
Nitrogen metabolism (N fixation)	18	17%
Phosphate metabolism	34	14%
Carbohydrate metabolism	34	26%
Photosynthesis	24	62%

**FIGURE 7:**
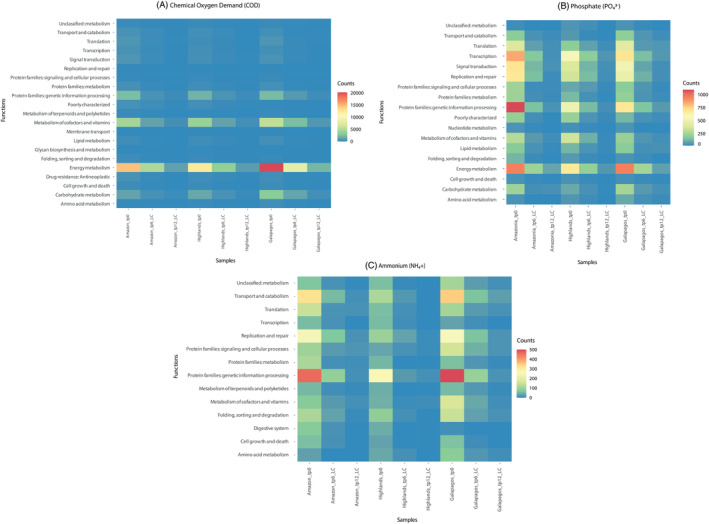
Heatmaps showing the raw abundance (counts) of the KP IDs collapsed into metabolic functions at a first‐order (based on the annotation according to the KEGG database) of three microalgae–bacteria consortia from the Ecuadorian Amazon, Highlands, and Galapagos regions incubated in SWW under light conditions (LC). Each heatmap is related to a specific nutrient or compound removed: (A) COD, (B) Phosphate (PO_4_^3−^), and (C) Ammonium (NH_4_^+^).

We employed an untargeted approach to investigate the abundance profiles of predicted genes exhibiting a strong correlation (*r* ^2^ > 0.7) with NH_4_^+^, PO_4_^3−^, and COD removal patterns. This analysis revealed 666 predicted genes common to all consortia, the majority of which were associated to first‐order fundamental metabolic processes such as replication, genetic information processing, cellular growth, signalling, and general metabolic activities (e.g., energy metabolism, lipid metabolism, carbohydrate metabolism, etc.). A prevailing trend observed across the three consortia for the identified predicted genes was a peak in raw abundance on D0, followed by a marked decline on D6, and minimal to no predicted activity by D12 (Figures 7 and S3). In general, the Amazon and Galapagos consortia exhibited higher abundance profiles on D0 compared with the Highlands consortium. Notably, for PO_4_^3−^ and COD, both the Amazon and Galapagos consortia displayed an appreciably higher enrichment of predicted genes associated with first‐order energy metabolism. Upon closer examination of these predicted genes at a second‐order metabolic level, it became evident that they were primarily associated with photosynthesis.

We conducted a taxonomic analysis to identify the microbial genera responsible for the predicted gene functions (from our targeted and untargeted analyses) linked to nutrient and organic matter removal. Overall, the three consortia exhibited distinct genera contributing to key predicted gene functions (Table 4). Specifically, for the Galapagos consortium, Sphingomonas emerged as the predominant genus contributing to the removal of NH_4_^+^, PO_4_^3−^, and COD, while for the Highlands and Amazon consortia, Shinella and Pedobacter were the dominant groups, respectively. Regarding photosynthesis, Parachlorella and Sphingomonas were predominant in the Galapagos consortium, Pedobacter and Chlorella in the Amazon consortium, and Shinella and Sphingopyxis in the Highlands consortium. Notably, Flavobacterium played a significant role in both the Amazon and Highlands consortia in nutrient and organic matter removal from SWW. However, it did not emerge as predominant group in the Galapagos consortium.

**TABLE 4:** Relative abundances (%) of the three most predominant bacterial genera related to ammonium removal, phosphate removal, COD, and photosynthesis for the three Ecuadorian microalgae–bacteria consortia from the Ecuadorian Amazon, Highlands, and Galapagos regions incubated in SWW.

Metabolism	Genus	Amazon	Genus	Highlands	Genus	Galapagos
Ammonium removal (NH4+)	*Pedobacter* spp.	15–34	*Shinella* spp.	32–57	*Sphingomonas* spp.	16–83
	*Sphingopyxis* spp.	21–32	*Sphingopyxis* spp.	18–23	*Parachlorella* spp.	6–69
	*Hydrogenophaga* spp.	15–31	*Flavobacterium* spp.	5–10	*Sphingopyxis* spp.	3–15
Phosphate removal (PO43‐)	*Pedobacter* spp.	18–48	*Shinella* spp.	31–61	*Sphingomonas* spp.	51–92
	Sphingopyxis spp.	13–19	*Sphingopyxis* spp.	11–18	*Chlorella* spp.	1–24
	*Hydrogenophaga* spp.	12–25	*Flavobacterium* spp.	9–15	*Sphingopyxis* spp.	3–16
COD	*Pedobacter* spp.	18–47	*Shinella* spp.	36–64	*Sphingomonas* spp.	47–91
	*Sphingopyxis* spp.	15–22	*Sphingopyxis* spp.	11–23	*Sphingopyxis* spp.	3–19
	*Flavobacterium* spp.	5–11	*Flavobacterium* spp.	6–12	*Devosia* spp.	2–10
Photosynthesis	*Pedobacter* spp.	5–42	*Shinella* spp.	14–38	*Parachlorella* spp.	2–12
	*Chlorella* spp.	12–35	*Sphingopyxis* spp.	21–30	*Sphingomonas* spp.	7–73
	*Sphingopyxis* spp.	9–21	*Flavobacterium* spp.	10–27	*Chlorella* spp.	18–80

## DISCUSSION

### Wastewater remediation potential of native consortia

The three microalgae‐bacteria consortia collected from the Amazon, Highlands, and Galapagos regions of Ecuador exhibited the capacity to remove organic matter and nutrients from SWW under light (LC) and continuous dark (CDC) conditions. In terms of overall performance, the Amazon and Galapagos consortia outperformed the Highlands consortium for the removal of NH_4_^+^, PO_4_^3−^, and COD. The bulk removal efficiencies observed in this study for PO_4_^3−^ and COD under LC for the Amazon and Galapagos consortia align with findings reported in literature. For example, Fan et al. (2020) achieved bulk removal efficiencies of 96% for PO_4_^3−^ and 88% for COD using a *Chlorella sorokiniana*‐activated sludge (AS) consortium cultivated in SWW. Similarly, a consortium of *C. vulgaris* and AS achieved bulk removal efficiencies of 39% for PO_4_^3**−**^ and 98% for COD in SWW (Corpuz et al., 2021), while another study reported up to 99% bulk removal of total phosphorous and 87% removal of COD from SWW using a consortium of *Chlorella* sp. and AS (Guo et al., 2023). In the case of NH_4_^+^, bulk removal efficiencies of microalgae co‐cultivated with AS ranged from 81% to 99.2% (Corpuz et al., 2021; Fan et al., 2020; Guo et al., 2023). These values are twice as high as bulk removal efficiencies registered in this study for the Amazon and Galapagos consortia. It is important to highlight that in the aforementioned studies, microalgae were purposely co‐cultivated with AS which had been conditioned to enhance the degradation of organic matter and the uptake of nutrients from wastewater. In this study, the Amazon and Galapagos consortia demonstrated robust PO_4_^3−^ and COD remediation capabilities without the need for AS. However, it is well‐documented that co‐cultivation with AS can enhance nitrogen removal due to the presence of specialized microbial communities involved in ammonium degradation, nitrification, and denitrification processes (Dueholm et al., 2022; Wang et al., 2016; Wu et al., 2019).

As expected, all three consortia demonstrated superior removal efficiencies under LC compared with CDC (Figure 2). Previous studies have shown that the co‐cultivation of microalgae and bacteria in SWW can result in enhanced nutrient removal capabilities, particularly in the presence of light (Cerón García et al., 2005; Cho et al., 2015; Fan et al., 2020; Perez‐Garcia et al., 2010). Light promotes microalgal autotrophic growth, which in turn stimulates the overall metabolic activity of the consortium through metabolic synergies with heterotrophic bacteria (Cerón García et al., 2005; Fan et al., 2020; Perez‐Garcia et al., 2010). Nevertheless, it is noteworthy that all three evaluated consortia achieved COD removal efficacies ranging from 85% to 88% of the values achieved under LC. This suggests that COD removal primarily relies on the heterotrophic machinery of the consortium and may be less dependent on metabolic synergies created through the autotrophic growth of microalgae under light. It is also worth highlighting that the Amazon consortium exhibited a 1.3‐ to 1.5‐fold superior performance in PO_4_^3−^ removal under CDC compared with the Galapagos and Highlands consortia under the same conditions. This distinct advantage of the Amazon consortium underscores the potential of bioprospecting to unveil consortia or specific organisms with heterotrophic (or mixotrophic) metabolic capabilities enabling the maximization of nutrient in closed‐wastewater systems or facilitating efficient wastewater reactor designs under light‐limited conditions.

### Effect of microbial diversity and composition in nutrient removal from wastewater

Recent metagenomic studies have emphasized the significant biodiversity and distinct community structures observed in microbial communities from wastewater treatment plants. Deterministic factors, such as geography, temperature, and process type, can shape these communities (Dueholm et al., 2022; Wu et al., 2019). Our β‐diversity and PERMANOVA analyses revealed notable genetic distancing among the three consortia based on their geographic origin (Figure 5). Additionally, the degree of α‐diversity (Inverse Simpson, Evenness) varied among the three consortia, with the Galapagos consortium displaying lower species biodiversity compared with the Amazon and Highlands consortia (Tables 2 and S2). However, we found that the extent of species diversity within the three consortia could not be directly or qualitatively linked to differences in their ability to remove nutrients and organic matter from SWW. This finding aligns with previous reports suggesting that α‐diversity and β‐diversity do not serve as primary drivers of the remediating capacity of wastewater remediating consortia (Dueholm et al., 2022; Wu et al., 2019). Instead, these reports propose that wastewater remediating microbial communities are enriched with core taxonomic groups known for their nutrient recovery, transformation, and removal functions. It is suspected that the presence and abundance of these specific core taxonomic groups serve as the primary drivers of consortium remediation capabilities (Dueholm et al., 2022; Wu et al., 2019).

In line with the above, we investigated whether the taxonomic composition of the evaluated consortia could be linked to differences in wastewater remediation capacity. Proteobacteria and Bacteroidetes were the most abundant bacterial phyla observed across the three consortia at the phylum level (Figure 3). These phyla have been highlighted in recent metagenomic studies as signature groups dominating microbial communities specialized in wastewater remediation (Dueholm et al., 2022; Moloantoa et al., 2023; Oluseyi Osunmakinde et al., 2019; Shanks et al., 2013; Wu et al., 2019). However, the relative abundance of these phyla did not correlate with the remediation efficacy of the consortia. For instance, the Highlands consortium had higher relative abundances of Proteobacteria and Bacteroidetes compared with the Amazon and Galapagos consortia but showed lower nutrient and organic matter bulk removal efficiencies. Notably, a key distinction between the Amazon and Galapagos consortia compared with the Highlands consortium was a significantly higher representation of Chlorophyta, or green algae. Under LC and CDC, the Amazon and Galapagos consortia consistently displayed higher Chlorophyta abundance over time, particularly at D0 (~36% Amazon, ~57% Galapagos, ~7% Highlands). The symbiotic relationship between microalgal and bacterial communities has been shown to enhance nutrient uptake, removal, and transformation in wastewater (Cerón García et al., 2005; Cho et al., 2015; Fan et al., 2020; Perez‐Garcia et al., 2010). Considering the marked increase in nutrient and organic matter bulk removal from D0 to D6, there is a possibility that the initial populations of Chlorophyta at D0 in the Amazon and Galapagos consortia influenced the metabolism of the consortium community, promoting the growth of heterotrophic bacteria and thus enhancing organic carbon removal and nutrient uptake relative to the Highlands consortium (Cho et al., 2015). However, it is important to note that the relative abundance of Chlorophyta decreased for all three consortia from D0 to D12 under LC and CDC. This decrease might indicate competition between microalgae and heterotrophic bacteria due to rapid nutrient and organic matter depletion, resulting in the growth stagnation of green algae and a generalized slowdown of the community's metabolism for PO_4_^3−^ and COD—as observed for the three consortia from D6 to D12 (Figure 2). Importantly, the Galapagos consortium exhibited an increase in Chlorophyta abundance from D6 to D12 under CDC. Previous studies have demonstrated that green microalgae can operate in mixotrophic or heterotrophic mode, utilizing carbon and nitrogen synergistically, with light acting as an accelerator for nutrient uptake (Cerón García et al., 2005; Cho et al., 2015; Perez‐Garcia et al., 2010). In this regard, the increase in the relative abundance of Chlorophyta seen in the Galapagos consortium in the absence of light could indicate the ability of the species of this genus to adopt heterotrophic growth (Amaro et al., 2023; Su et al., 2011).

At the genus level, a focused analysis of the most enriched taxonomic groups for the three consortia (Figure 4) reveals the occurrence of several microbial genera commonly associated with nitrogen metabolism (e.g., Xanthobacteraceae, Comamonas; Chlorella), polyphosphate accumulation (e.g., β‐proteobacteria), and organic matter degradation (e.g., Burkholderiales, Methylobacteriacea) in wastewater treatment (WWT) plants (Dueholm et al., 2022; Meng et al., 2016; Moloantoa et al., 2023; Tikhonova et al., 2021; Tsagkari & Sloan, 2019; Wu et al., 2019). These taxonomic groups were considerably more abundant in the Amazon and Galapagos consortia (~65% of all identified groups with LDA > 2) than in the Highlands consortium (~27% of taxonomic groups with LDA > 2). The lower abundance of specialist microorganisms known to participate in wastewater remediation and purification activities within the Highlands consortium could potentially explain the consortium's lower nutrient bulk removal efficiency (Dueholm et al., 2022; Moloantoa et al., 2023; Oluseyi Osunmakinde et al., 2019; Shanks et al., 2013; Wu et al., 2019).

Finally, although all three consortia included microorganisms commonly found in specialist wastewater microbial communities, they lacked key taxonomic groups that have been previously reported as part of “global core wastewater communities” (Dueholm et al., 2022; Wu et al., 2019). These key taxonomic groups include *Nitrosomonas* sp. and Nitrospiraceae involved in nitrification, *Haliangium* sp. involved in denitrification, Xanthomonadaceae involved in organic matter degradation, as well as *Tetrasphaera* sp. and *Acinetobacter* sp., involved in phosphate uptake (Dueholm et al., 2022; Rahayu, 2019; Stephenson, 1987; Wu et al., 2019). In particular, the absence of crucial nitrifiers could explain the significantly lower ammonium removal rates observed in this study (Corpuz et al., 2021; Fan et al., 2020; Guo et al., 2023). Ultimately, the absence of key taxonomic groups may be attributed to the fact that the three consortia used in this study were collected from freshwater reservoirs rather than wastewater treatment plants. Systematic exposure of these consortia to wastewater could lead to the emergence of the aforementioned key groups, considering their prevalence in global wastewater microbial communities (Dueholm et al., 2022; Wu et al., 2019).

### Functional profiles related to wastewater remediation capabilities

Identifying KEGG pathway enrichment patterns that could explain differences in nutrient bulk removal performance between the three consortia proved challenging, with a few exceptions. For instance, the Amazon and Galapagos consortia, which consistently demonstrated superior NH_4_^+^ bulk removal efficiencies compared with the Highlands consortium, exhibited a steady increase for the ammonium metabolism KP ID K03320 from D0 to D12 under both LC and CDC conditions (Figure 6). In contrast, the Highlands consortium showed a decrease in the abundance of KP ID K03320 over the same period under both conditions. The relative abundance of the most abundant KP ID for nitrogen fixation (K13598) declined from D6 to D12 under LC and CDC for the Galapagos and Highlands consortia but increased for the Amazon consortium during the same period (Figure 6). While the Amazon and Galapagos consortia displayed similar removal patterns for NH_4_^+^ (i.e., sharp removal efficacy from D0‐D6, followed by qualitatively lower removal efficiencies from D6 to D12), a potential increase in the nitrogen fixation activity of Amazon consortium could explain its comparatively higher removal efficiencies from D6 to D12 under LC (21% higher for NH_4_^+^) and CDC (11% higher for NH_4_^+^) (Figure 2) (Corpuz et al., 2021; Fan et al., 2020; Guo et al., 2023). On a broader spectrum, we also discovered that for PO_4_^3−^ and COD, the Galapagos and Amazon consortia exhibited markedly higher enrichment of genes associated with first‐order energy metabolism, specifically linked to increased photosynthetic metabolic activity (Figure 7). This observation is consistent with the higher representation of Chlorophyta observed in the Amazon and Galapagos consortia on D0 compared with the Highlands consortium. As previously established, microalgal autotrophic growth has been demonstrated to enhance the metabolic capacity of microalgae–bacteria consortia, thereby improving nutrient uptake, removal, and transformation in wastewater (Cerón García et al., 2005; Cho et al., 2015; Fan et al., 2020; Perez‐Garcia et al., 2010). This could suggest that the increased presence of autotrophic microalgae in the Amazon and Galapagos consortia—and their cooperative metabolic interactions with co‐cultivated bacteria—on D0 could partially explain their heightened metabolism for PO_4_^3−^ and COD compared with the Highlands consortium.

We identified several taxonomic groups potentially responsible for predicted gene functions associated with the removal of nutrients and organic matter in SWW (Table 4). From these, Chlorella (Amazon), Sphingomonas (Galapagos), Shinella (Highlands), and Sphingopyxis (Highlands) were also among the most differentially abundant taxa for their respective consortia (Figure 4). This was not the case for other groups such as Pedobacter (Amazon), Parachlorella (Galapagos), and Flavobacterium (Amazon and Highlands). From the listed genera above, only Chlorella and Flavobacterium have been previously reported as signature groups dominating microbial communities specialized in wastewater remediation (Dueholm et al., 2022; Moloantoa et al., 2023; Oluseyi Osunmakinde et al., 2019; Shanks et al., 2013; Wu et al., 2019). Although the other identified taxonomic groups have not been reported as part of the core microbial groups commonly found in wastewater communities, different studies have shown that these organisms have versatile metabolisms that can contribute to wastewater remediation. For instance, Sphingomonas and Shinella have been reported as genera involved in nitrogen fixation (Alok et al., 2020; Bizjak et al., 2023), phosphate fixation (Asaf et al., 2020; Jin et al., 2020; Liu et al., 2019), carbohydrate metabolism (Anderson & Wood, 1969), and photosynthesis (Kopejtka et al., 2021; Lu et al., 2022). Furthermore, while Pedobacter is a less studied genus, it has been associated with nitrogen fixation and phosphate fixation (Liang et al., 2022; Vannier et al., 2023); and Parachlorella and the bacterial genus Sphingopyxis are recognized for their ability to participate in photosynthesis (Kopejtka et al., 2021; Sharma et al., 2021; Vello et al., 2018). Several studies, including those by Dueholm et al. (2022) and Wu et al. (2019), have demonstrated a connection between taxonomy and function within wastewater microbial communities. However, our findings suggest that the metabolic capacity of the consortium is primarily influenced by the presence or absence of specific metabolic capabilities rather than the taxonomic composition of the consortium. Louca et al. (2016) have shown that environmental conditions play a pivotal role in shaping the metabolic functions of microbial communities by delineating metabolic niches that can accommodate a diverse array of functionally similar microbial species. Presumably, this phenomenon is facilitated by the high degree of functional redundancy inherent in global microbial communities (Louca et al., 2016).

Previous studies have demonstrated that co‐culturing microalgae and bacteria generally leads to higher nutrient removal rates, particularly in the presence of light, which promotes the growth of microalgae and stimulates overall community metabolism through metabolic synergism (Cerón García et al., 2005; Cho et al., 2015; Fan et al., 2020; Perez‐Garcia et al., 2010). In this study, nutrient bulk removal efficiencies were higher under LC than CDC for all three consortia, but we could not detect higher metabolic activity (i.e., expressed as relative abundance) for photosynthesis or carbon metabolism pathways under LC relative to CDC. Moreover, it should be noted that for the Galapagos consortium, the relative abundance of Chlorophyta was higher under CDC than LC. This finding shows that the co‐cultivation of microalgae and bacteria is not always a driver of improved nutrient removal efficacy, and that other factors may play a role in increasing or diminishing the community's wastewater remediating capacity. It has been suggested, for example, that a lack of light generates oxidative stress in microalgae–bacteria consortia under dark conditions, which in turn reduce the metabolic performance of the metabolic community (Fan et al., 2020). This emphasizes the importance of understanding the unique functionalities of different microbial communities, as deeper insights may provide clues about specific microorganisms or conditions that can be utilized to modify, enhance, or optimize remediation processes. Exploring native consortia could provide valuable metabolic functions including interesting microorganisms such as facultative mixotrophic algae to improve bioremediation processes.

## AUTHOR CONTRIBUTIONS

**Juan José Guadalupe:** Data curation (equal); formal analysis (equal); investigation (equal); methodology (equal); visualization (equal); writing – original draft (equal). **Miguel Pazmiño‐Vela:** Data curation (equal); formal analysis (equal); investigation (equal); software (lead); visualization (equal); writing – original draft (equal). **Gabriela Pozo:** Data curation (equal); formal analysis (equal); investigation (supporting); methodology (equal); writing – original draft (equal). **Wendy Vernaza:** Data curation (equal); formal analysis (equal); investigation (supporting). **Valeria Ochoa‐Herrera:** Conceptualization (supporting); methodology (equal); project administration (supporting); resources (supporting); writing – original draft (supporting); writing – review and editing (supporting). **Maria de Lourdes Torres:** Conceptualization (supporting); methodology (equal); resources (lead); supervision (supporting); writing – review and editing (supporting). **Andres F. Torres:** Conceptualization (lead); data curation (equal); formal analysis (supporting); funding acquisition (lead); methodology (equal); project administration (lead); supervision (lead); validation (equal); writing – original draft (equal); writing – review and editing (equal).

## CONFLICT OF INTEREST STATEMENT

The authors declare that there is no conflict of interest.

## Supporting information


**Data S1.** Supporting Information Figure.

**Data S2.** Supporting Information Tables.

## Data Availability

The data that support the findings of this study area available from the corresponding author upon reasonable request.
