# Supplementary material for: Metagenomic analysis of microbial consortia native to the Amazon, Highlands, and Galapagos regions of Ecuador with potential for wastewater remediation
Source: Environ Microbiol Rep. 2024 May 1;16(3):e13272. doi: 10.1111/1758-2229.13272 (PMC11062868; doi:10.1111/1758-2229.13272)
Supplement: Supplementary file 1 — Data S1. Supporting Information Figure. [file EMI4-16-e13272-s002.docx]

**SUPPLEMENTARY MATERIAL**

**FIGURES**

**
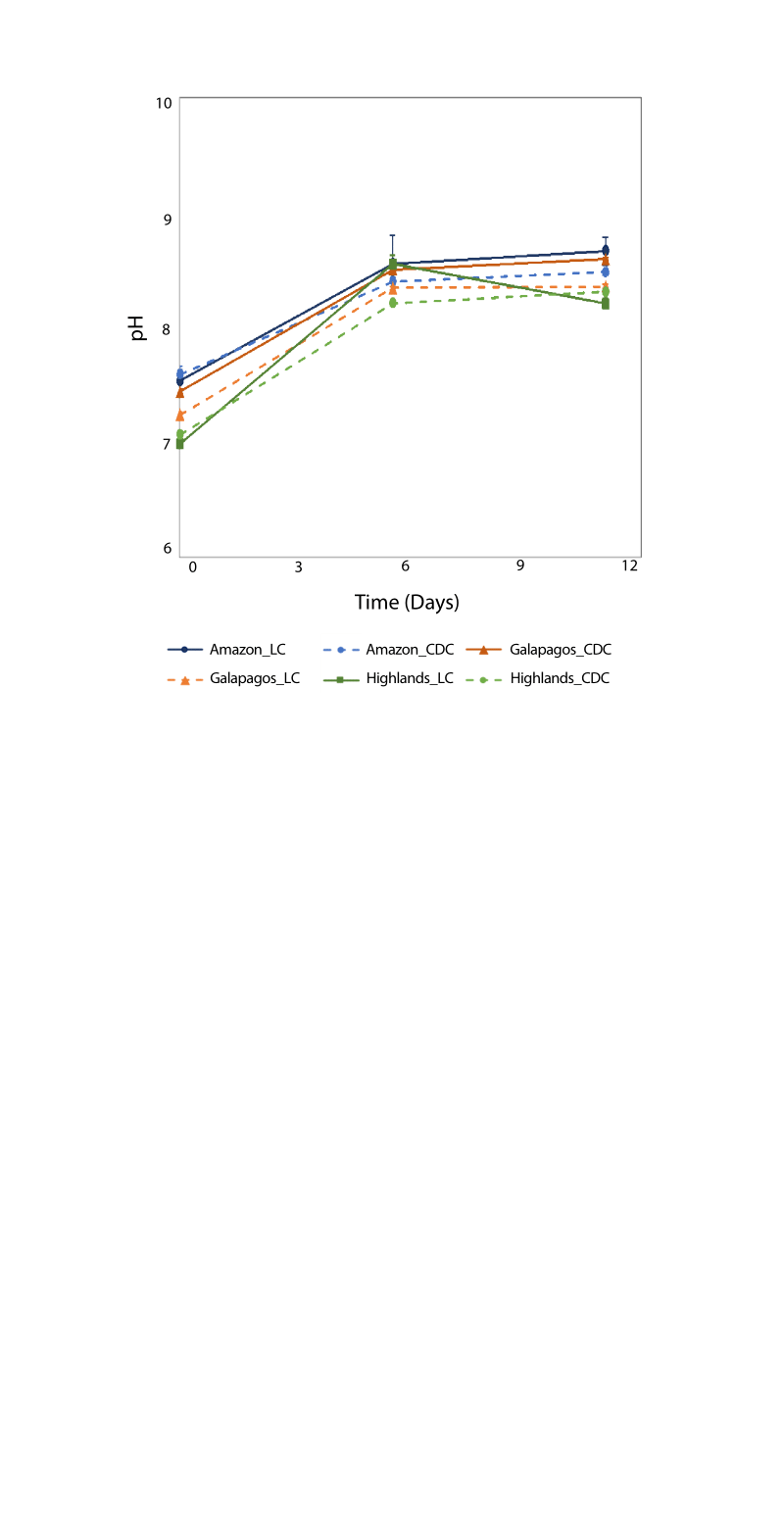
**

**Supplementary Figure 1.** Progression of pH during nutrient removal assays for three Ecuadorian microbial consortia from the Amazon, Highlands, and Galapagos regions incubated in synthetic wastewater (SWW) under light (LC) and continuous dark (CDC) conditions.


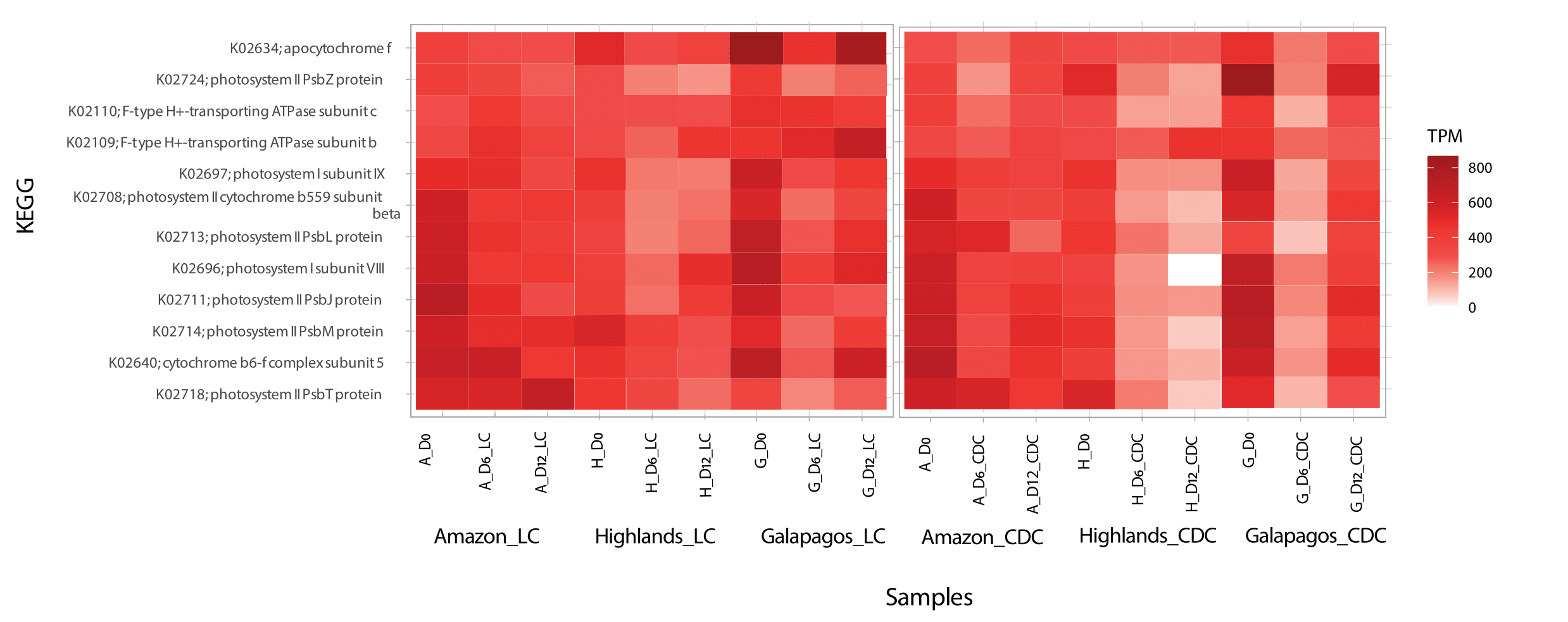


**Supplementary Figure 2.-** Heatmaps showing the relative abundance of the 12 most abundant Photosynthesis related KEGGs identified for each of the Algae-Bacteria consortia at three points in time exposed to two different treatments. TPM (transcripts per million) refers to the number of genes from each KEGG pathway per million genes in the metagenome.


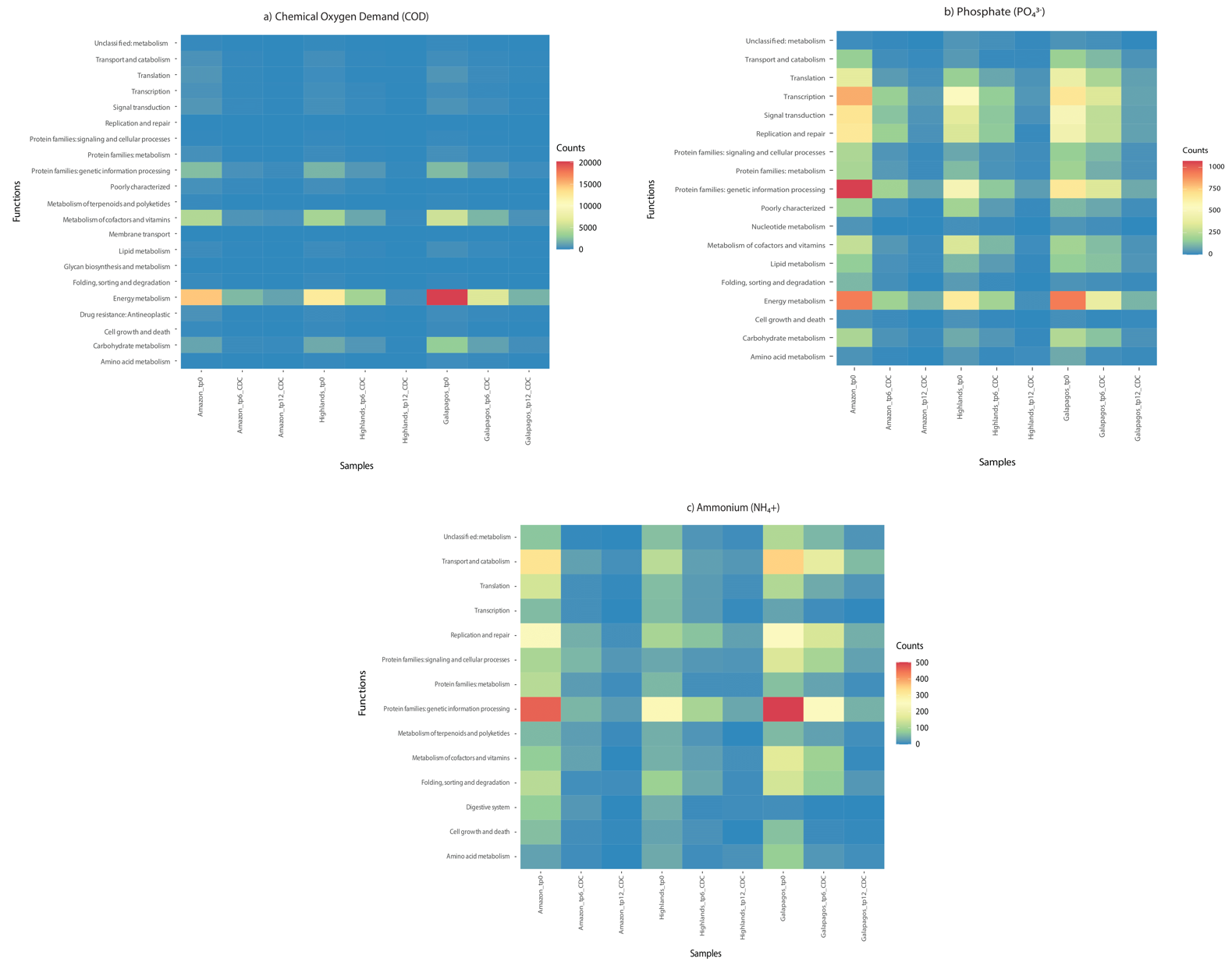


**Supplementary Figure 3.-** Figure 7. Heatmaps showing the raw abundance (counts) of the KP IDs collapsed into metabolic functions at a first-order (based on the annotation according to the KEGG database) of three micro-algae bacteria consortia from the Ecuadorian Amazon, Highlands and Galapagos regions incubated in synthetic wastewater (SWW) under continuous dark conditions (CDC). Each heatmap is related to a specific nutrient or compound removed: a) Chemical oxygen demand, b) Phosphate (PO43-), and c) Ammonium (NH4+).
