# Supplementary material for: Metagenomic analysis of microbial consortia native to the Amazon, Highlands, and Galapagos regions of Ecuador with potential for wastewater remediation
Source: Environ Microbiol Rep. 2024 May 1;16(3):e13272. doi: 10.1111/1758-2229.13272 (PMC11062868; doi:10.1111/1758-2229.13272)
Supplement: Supplementary file 2 — Data S2. Supporting Information Tables. [file EMI4-16-e13272-s001.docx]

**SUPPLEMENTARY MATERIAL**

**TABLES**

**Supplementary Table 1.** Nanopore sequencing output details for three algae-bacteria consortia from the Amazon, Highlands and Galapagos regions of Ecuador incubated in SWW for a 12-day period (D_0,_ D_6,_ D_12_) under light (LC) and continuous dark (CDC) conditions.

|  |  | **Treatment and Timepoint** | | | | |
| --- | --- | --- | --- | --- | --- | --- |
| **Consortia** | **Parameter** | **LC/CDC D_0_** | **LC D_6_** | **LC D_12_** | **CDC D_6_** | **CDC D_12_** |
| **Amazon** | Mean read length | 1521.6 | 922.3 | 999.0 | 995.3 | 820.6 |
|  | Mean read quality | 11.1 | 11.4 | 11.3 | 10.6 | 10.6 |
|  | Read length N50 | 4874.0 | 2227.0 | 2966.0 | 2029.0 | 1264.0 |
|  | Q7 (%) | 100 | 100 | 100 | 99.9 | 99.9 |
| **Galapagos** | Mean read length | 813.0 | 2950.8 | 1948.9 | 1287.3 | 2062.9 |
|  | Mean read quality | 11.1 | 11.4 | 11.4 | 10.6 | 10.7 |
|  | Read length N50 | 1196.0 | 5787.0 | 4246.0 | 3212.0 | 7509.0 |
|  | Q7 (%) | 100 | 100 | 100 | 99.9 | 100 |
| **Highlands** | Mean read length | 2739.2 | 1975.5 | 2266.7 | 1895.3 | 1873.2 |
|  | Mean read quality | 11.0 | 13.4 | 13.2 | 10.7 | 10.7 |
|  | Read length N50 | 6612.0 | 6067.0 | 6583.0 | 5203.0 | 5365.0 |
|  | Q7 (%) | 100 | 100 | 100 | 100 | 100 |

**Supplementary Table 2.** α-diversity indices at different timepoints for three Ecuadorian microalgae-bacteria consortia from the Amazon, Highlands and Galapagos regions incubated in SWW for a 12-day period under continuous dark (CDC) conditions.

|  |  | **α-diversity Index** | | |
| --- | --- | --- | --- | --- |
| **Consortia** | **Timepoint** | **Shannon** | **Inverse Simpson** | **Evenness** |
| **Amazon** | D_0_ | 2.35 | 6.41 | 0.57 |
|  | D_6_ | 2.58 | 9.62 | 0.62 |
|  | D_12_ | 2.61 | 9.59 | 0.64 |
| **Galapagos** | D_0_ | 1.85 | 4.31 | 0.46 |
|  | D_6_ | 1.57 | 2.31 | 0.39 |
|  | D_12_ | 2.01 | 3.82 | 0.5 |
| **Highlands** | D_0_ | 2.32 | 5.43 | 0.54 |
|  | D_6_ | 2.31 | 5.51 | 0.55 |
|  | D_12_ | 2.35 | 5.56 | 0.57 |
